# Supplementary material for: Patterns of Intron Gain and Loss in Fungi
Source: PLoS Biol. 2004 Nov 30;2(12):e422. doi: 10.1371/journal.pbio.0020422 (PMC532390; doi:10.1371/journal.pbio.0020422)
Supplement: Table S1 — Also available at http://genes.mit.edu/NielsenEtAl/. (4.3 MB ZIP). [file pbio.0020422.st001.zip › NielsenEtAl/html/1249.html]

AN0630.1.NCU00160.1.MG05679.1.FG02535.1


```
 CLUSTAL W (1.82) Multiple Sequence Alignments - Introns Inserted


Sequence 1: MG05679.1	67 aa
Sequence 2: FG02535.1	72 aa
Sequence 3: NCU00160.1	55 aa
Sequence 4: AN0630.1	84 aa
Alignment Length: 84 aa
Number Identitical Residues: 25 aa
Alignment Score (without introns) 1467


MG05679.1 	-----~--~MSLDPAIVKL--~--------GH1MNTNRYKYFRWTPRTVKISLMYVVVVP
NCU00160.1	-----~--~------------~----------~MTNNRYKYFRWTPRTAGITFAYMVAVP
FG02535.1 	MAHLN1--~VKPDPAYLKY--~--------AA1MMKSRHHYFRWTPRTAKITFIYVGVIP
AN0630.1  	MAGPS~KS1LILDPALQKYYV1DLTNAWAELE~VNSNRYKYFRWTPRTAWHSLLYMVLIP
          	 :  . .:    ..:  .    . :.: :.   : ..*::********.  :: *:  :*

MG05679.1 	SILGYFAYTTD0GKWDFRAKRKGDDIREF
NCU00160.1	GILGYIAYKTD0GKYDFRAKRRGDDMREY
FG02535.1 	AIMGYIAYKTD0GLWDFRAKRKGDLVYEK
AN0630.1  	ASLGYVAYKSD0GKYDFRGKRRGDTIAEW
          	. :**.**.:* * :***.**:** : *
```
